# Influence of Particle Size and Bulk Density on Sound Absorption Performance of Oil Palm Frond-Reinforced Composites Particleboard

**DOI:** 10.3390/polym15030510

**Published:** 2023-01-18

**Authors:** Budi Istana, I Made Londen Batan, Sutikno, Samrith Khem, U Ubaidillah, Iwan Yahya

**Affiliations:** 1 Department of Mechanical Engineering, Institut Teknologi Sepuluh Nopember (ITS), Surabaya 60111, Indonesia; 2 Department of Mechanical Engineering, Universitas Muhammadiyah Riau (UMRI), Pekanbaru 28294, Indonesia; 3 Department of Mechanical Engineering, Universitas Sebelas Maret (UNS), Surakarta 57126, Indonesia; 4 Department of Physics, Universitas Negeri Sebelas Maret (UNS), Surakarta 57126, Indonesia

**Keywords:** oil palm frond, composite, particleboards, acoustic absorber, sound absorption coefficient

## Abstract

The present study deals with the sound absorption performance of natural fibres from the oil palm frond (OPF), mainly considered agricultural waste. Therefore, this study aimed to investigate the sound absorption performance of OPF fibre-reinforced composite under normal incidence sound. The materials used were OPF particles and urea-formaldehyde was used as an adhesive. The particleboards were produced with three particle sizes and four target densities. The absorption coefficient of normal incidence sound (α_n_) was tested using an impedance tube. The effects of particle size and bulk density were also evaluated. The findings reveal thatα_n_ exceeded 0.45 at 1000 Hz and could reach 0.95 above 3.3 kHz. This occurred when the bulk density of the OPF composite particleboards ranged between 0.3–0.4 g/cm^3^, and the particle size varied between medium to coarse. The results also indicated that the absorption frequency and the degree of α_n_ significantly increased as the bulk density decreased. Therefore, OPF fibres can be used to create sound-absorbing composite particleboards.

## 1. Introduction

Oil palm fronds (OPF) are one of Indonesia’s most abundantly available agricultural by-products. Since they are currently regarded as the waste product of oil palm fields, their biomass remains underutilised. The amount of OPF waste generated in the country has significantly increased from 85,488,280 tonnes in 2010 to 145,865,970 tonnes in 2020 and continues to increase, covering an area of approximately 14,586,597 hectares [1]. OPF are leftovers from post-harvesting and trimming, and the amount produced largely depends on the age of the palm tree. According to present estimates, approximately 10,400 kg/ha of this waste is produced annually [2]. Meanwhile, replanting activities are believed to yield roughly 14,500 kg/ha of OPF [3]. Since landowners often do not know how to properly dispose of felled OPF, they are frequently left as garbage or burned with no discernible purpose, adversely impacting the environment. As a result, the relevant authorities have to incur significant costs to clean and dispose of this waste in an environmentally safe manner.

The huge volume of discarded oil palm fronds has the potential to be utilised in non-structural wood-based industries [4,5]. Several investigations have been undertaken on oil palm wood, including the use of fronds in particleboards [4], plywood [6], and bio-composites [7,8]. The properties of oil palm fronds as acoustic absorber materials in structures, however, have not been much discussed. In order to produce sound insulation materials with effective sound absorption for noise control and apply them to conventional structures, it is necessary to determine their properties. Currently, engineering noise control measures, such as sound-absorbing materials, are used to reduce the level of exposure of at-risk groups. Mineral wool, acoustic tiles, open-cell foams, and glass fibres are natural and man-made fibrous or porous commercial absorbers. They often disperse sound energy inside their chambers through visco-thermal processes. These synthetic absorbers are extremely robust, long-lasting, less thermally conductive, reasonably fire retardant, and resistant to moisture absorption, including bacterial and fungal development [9]. Despite these advantages, there is a cost associated with using such absorbers and insulators. The manufacture and use of these synthetic materials pose substantial risks to human health and the environment’s safety, especially since they contribute to global warming [10].

Using natural alternatives, either alone or as a component of composite materials, provides significant benefits, particularly in terms of environmental concerns compared to synthetic fibres. Natural fibres are typically inexpensive, plentiful, and have low densities. Furthermore, they are a renewable resource and less abrasive to machinery, tools, and equipment; they also contribute to better CO_2_ absorption, reduced emissions of fumes and harmful gases during production or combustion, and less skin and respiratory irritation [11,12]. These features have made these materials a creative resource for developing thermal and acoustic insulation materials, particularly in the building sector of underdeveloped countries, where the absence of adequate recycling laws is a major problem.

Despite the benefits of using natural fibre, its use in industry is accompanied by disadvantages such as poor fire and water resistance, weak fibre matrix bonding, and reduced durability. Raw fibres’ surface and structural characteristics have been changed or improved through diverse procedures, including physical, mechanical, and chemical pre-treatments. Recent research has focused on using natural, recyclable, biodegradable and sustainable alternatives to synthetic acoustic absorbers to address these disadvantages.

Several parameters, including pre-treatment, thickness, particle size, and density, affect the quality of sound insulation material. According to previous research, variations in density and particle size have been confirmed as one method for enhancing the acoustic characteristics of materials. Sihabut et al. [13] reported that particleboard produced from palm fronds with varying densities significantly influences acoustic properties. The investigation revealed that the optimal density of fibreboard was 0.276 g/cm^3^ with a coefficient of 0.78. Table 1 summarizes some recent studies on the acoustic absorption of natural fibre materials.

OPF is an intriguing alternative acoustic material due to the sheer annual volume produced, the sound-absorbing qualities, and ecologically sustainable manufacturing and disposal methods [25]. Furthermore, the acoustic quality of its fibre is comparable to that of wood, which varies depending on the anatomical structure, density, moisture content, and environmental temperature. Despite the sound reflection of a dense wooden structure, it can easily transform into a surface that channels sound [26]. OPF fibres are readily available and easily obtained in Indonesia. This is a straightforward alternative to wood as it can be easily crushed into chips identical to fibres or particles. In addition, OPF fibres are also renewable, abundant, non-abrasiveness, cheap, and pose less of a health and safety risk during processing and handling. Lastly, since this is a naturally occurring fibrous material, it is a sustainable alternative to synthetic fibres in sound absorption particleboards.

This project aims to create acoustic materials from oil palm fronds and investigates the influence of particle size and bulk density on the sound-absorbing characteristics of these materials. We produced composites from oil palm fronds using urea-formaldehyde as the binder.

## 2. Materials and Methods

### 2.1. Materials

**Figure 1:**
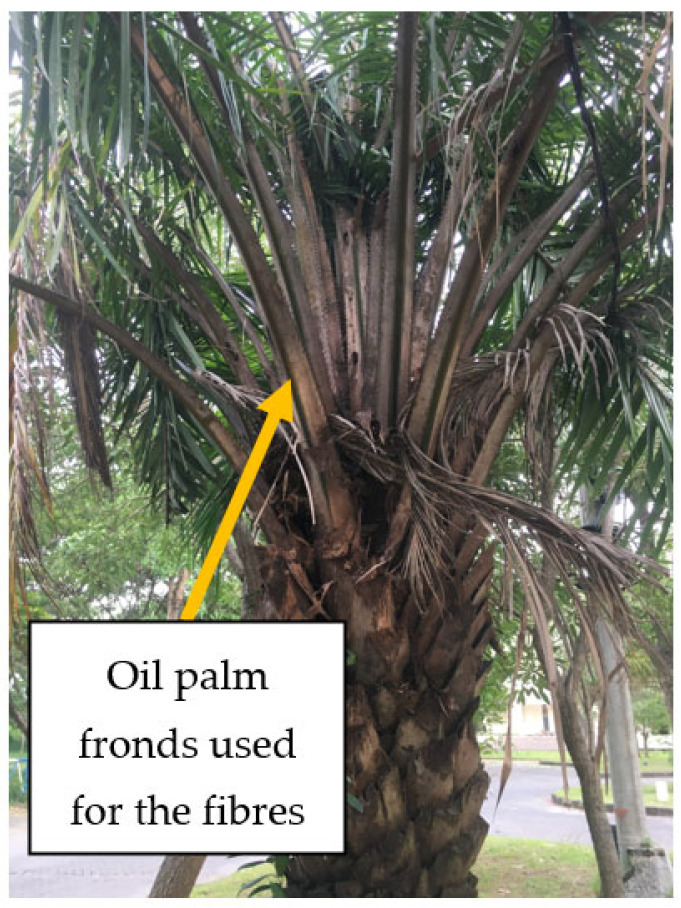
Oil palm tree in Riau, Indonesia.

**Figure 2:**
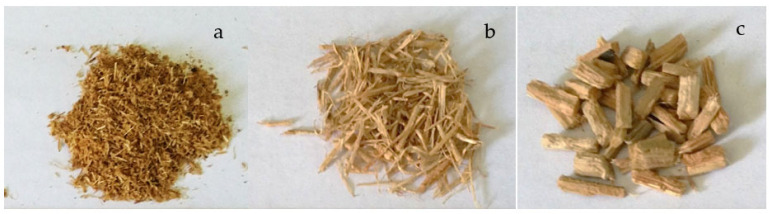
The OPF particles with different sizes (**a**) fine, (**b**) medium, (**c**) coarse.

Oil palm fronds aged about 20 years were cut and collected from a plantation in Riau, Indonesia, during the pruning process. Figure 1 shows the oil palm tree from which the fronds were utilised as the source of fibres for this study. The leaf blades were removed manually from the frond before sun-drying and ground into small particles ranging between 0.2–4.76 mm using a laboratory-made disc mill machine. The OPF particles were then screened and divided into fine, medium, and coarse particle sizes with dimensions ranging between 0.2–0.6 mm, 1.0–2.0 mm, and 2.38–4.76 mm, respectively (Figure 2). The approximate moisture content of the particles was 10%. As a binder, 10 wt.% (based on the weight of the particles) of urea-formaldehyde (UF) with a solid content concentration of 64.5%, a viscosity of 1800–2500 cPS at 30 °C, and a pH of 8.0–9.0 was used. Ammonium nitrate, at a concentration of 1.0 wt.% (based on the weight of the particles), was used as a hardener.

### 2.2. Preparations of Samples

**Figure 3:**
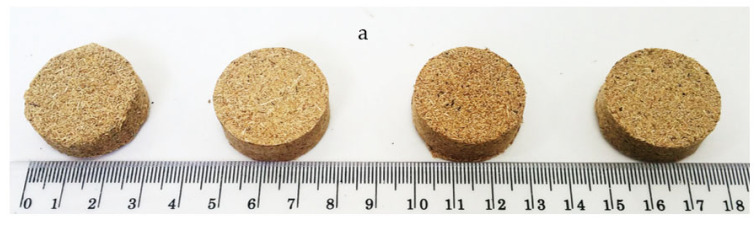

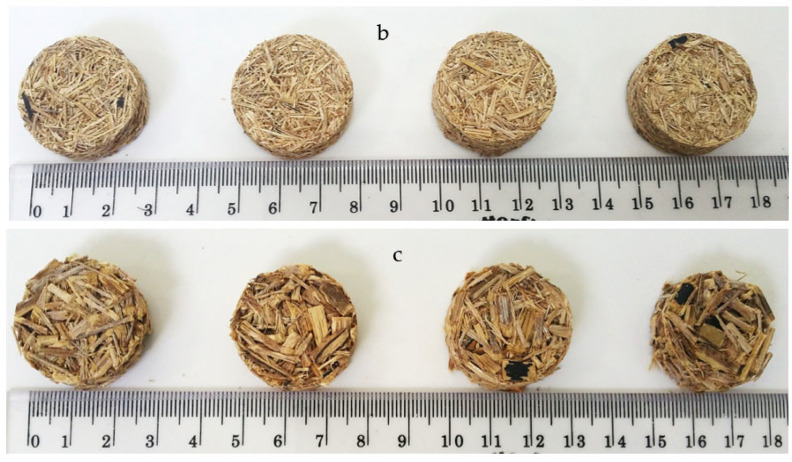
Acoustic test samples with different particle sizes and densities (**a**) fine, (**b**) medium, (**c**) coarse.

Single-layer samples measuring 29 mm in diameter and 10 mm in thickness were created at 0.3 g/cm^3^, 0.4 g/cm^3^, 0.5 g/cm^3^, and 0.6 g/cm^3^. To meet this set of densities, the mass of OPF fibres was measured accurately. Table 2 shows the type of board manufactured. A pneumatic nozzle was used to spray the UF resin onto the particles before a rotary drum mixer blended the mixture for five minutes at ambient temperature. Subsequently, the mixed particles were placed into separate stainless-steel pipe moulds measuring 29 mm in diameter and 200 mm in height for pre-pressing in a cold press, a process that reduced the thickness of the composites. The hot press was pre-set at 140 °C and pressed for five minutes at 1 MPa. The samples were stored in a conditioning room maintained at 20 ± 2 °C at 65% relative humidity. Finally, the purpose of conditioning was to enhance dimensional stability and surface quality and reduce formaldehyde emissions [27]. Figure 3 shows the created samples.

### 2.3. Measurement of Sound Absorption Coefficient (SAC)

**Figure 4:**
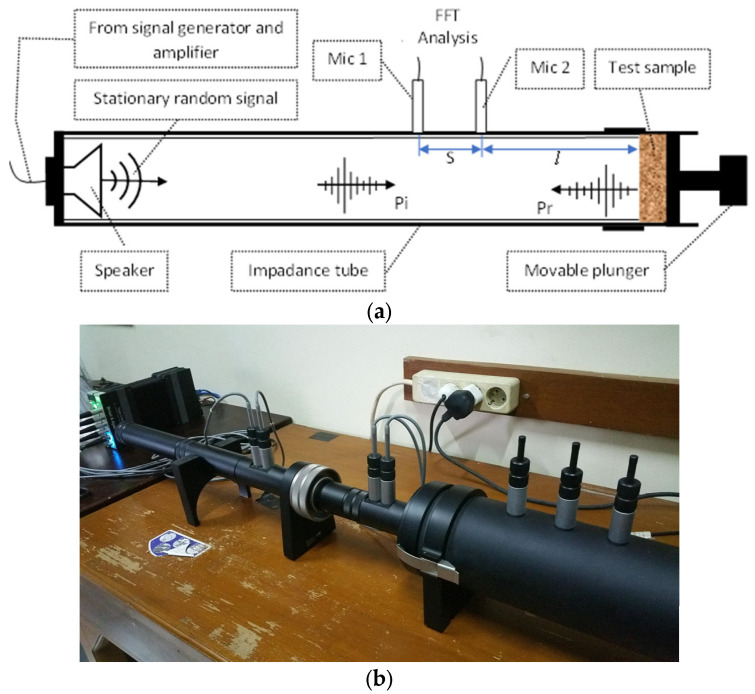
Acoustic sound absorption coefficient measurement: (**a**) Experimental setup for SAC Testing of the two-microphone impedance tube; (**b**) Measurement setup in the laboratory.

The normal incidence absorption coefficient was measured using an impedance tube [28]. Brüel and Kjær^®^ two-microphone impedance measurement tube type 4206 was used to calculate the normal α_n_ of the OPF composites following the transfer-function method [29]. Figure 4 depicts the experimental setup for measuring the sound absorption coefficients. The equipment in the impedance tube included two microphones, a loudspeaker, and a frequency analyser. A loudspeaker at one end of the tube created broadband random sound waves, which were subsequently transferred to the sample’s surface, fitted in a holder at the other end. Before calculating the normal incidence absorption coefficient, the reflected signals were acquired by microphones installed at two fixed positions on the tube wall. Finally, the data were processed using the PULSE LabShop software.

The samples with varying particle sizes and bulk densities were inserted into the holding tubes, and a rigid movable plunger could interchange their positions. The measurements were performed at least three times for each sample and the results were reported. In addition, the procedure of sample placement and rearrangement within the sample holder was performed for each sample in order to prevent and minimise the risk of mistakes resulting from improper sample placement. The experiments were conducted under controlled climatic conditions of (20 ± 2) °C and (45 ± 10) % relative humidity.

This approach uses the corrected acoustic transfer function (H12) to determine a test sample’s complex sound-reflection coefficient (R). The following is the complex sound reflection coefficient, according to Chung and Blaser [30]:

(1) $$R=\frac{H_{12}-e^{-jks}}{e^{jks}-H_{12}}e^{2jk\left(l+s\right)}$$

where *k* = 2πf/c is the wave number; *l* is the distance between microphone 2 and the front of the test sample; and *s* is the distance between the two microphones, as shown in Figure 4a. The normal incidence α_n_ and the specific impedance ratio (Z/c) were calculated using [30,31,32]:

(2) $$\frac{Z}{c}=\frac{1+R}{1-R}$$

(3) $$\alpha_{n}=1-{\left|R\right|}^{2}$$

where ρ and *c* are the density and speed of sound in the air, respectively. It should be noted that a normal α_n_ indicates a porous material that is capable of absorbing sound energy at different frequency bands.

## 3. Results and Discussion

### 3.1. Effect of Particle Size

**Figure 5:**
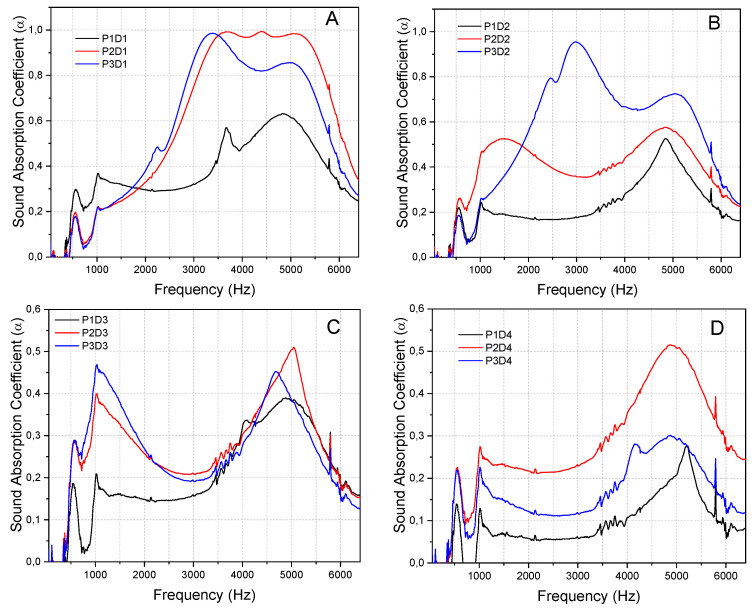
Sound absorption coefficients of the same density samples of OPF fibres with different particle sizes(**A**) 0.3 g/cm^3^, (**B**) 0.4 g/cm^3^, (**C**) 0.5 g/cm^3^, (**D**) 0.6 g/cm^3^.

According to Figure 5, fine particles provide less α_n_ than their medium and coarse counterpart within the parameters of this study.

The mechanism of sound energy dissipation in a porous material requires that the waves easily penetrate the material’s surface via its openings and pores, allowing its dissipation. Fine particles yield an OPF composite with a smoother surface, filling empty spaces, and sealing pores. Meanwhile, the medium and coarse particles produced OPF composites without a solid bond. This improved the porosity and tortuosity, enabling sound waves to move through it more quickly. Therefore, the α_n_ improved as viscous shear, friction, and structural vibrations resulted in a loss of incident energy.

According to Figure 5C,D, the α_n_ of the fine particle sample was low and did not exceed 0.4 when the frequency was below 4000 Hz. However, it was better than the medium and coarse samples when the frequency was below 1000 Hz (Figure 5A). It implies that fine particle composites are more reflective at below 4000 Hz. This is because the reflected energy increases and the sound dissipation reduces as the incident energy cannot penetrate the composite [33]. Figure 5A,B show that the α_n_ of the medium and coarse particle samples increased significantly at an α of more than 0.95 and a frequency of 700–3300 Hz. Furthermore, the α_n_ of medium and coarse particle samples with bulk densities of 0.5 and 0.6 g/cm^3^, respectively, did not exceed 0.53 throughout the entire frequency range (Figure 5C,D). This could be due to the various densities of the particleboards, indicating varying degrees of porosity. Consequently, particleboards with higher densities have a lower porosity that degrades their acoustic properties. The results of this study are consistent with those of other research reporting on the frequency-based acoustic performance of particleboard made from various plant fibres. This includes being excellent at low and high frequencies but poor at the medium level [34,35].

### 3.2. Effect of Bulk Density

**Figure 6:**
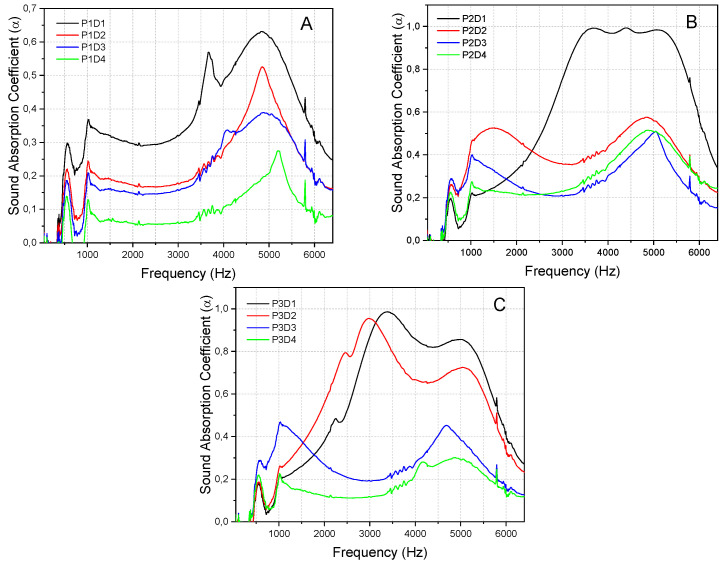
Sound absorption coefficients of the same particle size samples of OPF fibres with different densities. (**A**) small particle size, (**B**) Medium particle size, (**C**) Coarse particle size.

Density significantly affects the sound absorption performance of particleboards. This is because the bulk density of a fibrous material significantly affects its porosity, resulting in different sound absorption behaviours at different densities. For example, the densities are directly proportional to the energy loss as the absorber’s complexity of the sound path (tortuosity) increases. Therefore, this study analysed the α_n_ of four different densities of OPF composites on a single graph to better elucidate the effect of density on α_n_. Figure 6 compares the α_n_ of four reinforced composites of varying densities but the identical thickness and particle sizes.

The α_n_ decreased with increasing density for particles of identical size. Enhanced airflow resistivity caused a rise in α_n_ as density reduced because it restricted the sound path (tortuosity). Extant studies have also reported that the α_n_ decreases at higher densities [36]. At a density of 0.3 g/cm^3^, good α_n_ was obtained at all particle sizes, where α exceeded 0.3 above 2 kHz, which is the typical range of a fibrous absorber. The high normal incidence α_n_ was due to the high dissipation of the sound penetrating the material via viscous thermal interactions. The increase in porosity when density decreases causes the sound waves to penetrate the sample smoothly, enhancing the absorption performance of the low-density sample. An increase in density enhances tortuosity and enables more sound waves to be trapped or absorbed. A value that is too high can reduce the porosity of a sample and significantly increase its flow resistivity. This makes it difficult for sound waves to penetrate a material, reducing its absorption performance. According to Figure 6B, the α_n_ of samples with medium particle sizes and a density of 0.3 g/cm^3^ increased by more than 0.9 at high frequencies of 2700–5500 Hz. However, the α_n_ of samples with medium particle sizes was lower than the others at the initial stages, where the frequency was below 1300 Hz. Figure 6C shows that the α_n_ of samples with coarse particle sizes and densities of 0.3 and 0.4 g/cm^3^ steadily increased at 800–3000 Hz, with the highest α_n_ being more than 0.9. There is a higher increase in α_n_ at the 0.4 g/cm^3^ density sample than the 0.3 g/cm^3^ counterpart. Meanwhile, the α_n_ of the 0.3 and 0.4 g/cm^3^ density samples significantly decreased at 3500–6400 Hz. This reduction was higher in the 0.3 g/cm^3^ density sample. However, increasing the density of the samples to 0.5 and 0.6 g/cm^3^ resulted in an α_n_ of less than 0.47 and 0.3 across the entire frequency range, respectively. Therefore, the α_n_ decreased as the density increased due to high flow resistivity and tortuosity caused by an increase in fibre content.

### 3.3. Comparison with Commercial Wood

Table 3 describes α_n_ based on standardised octave band centre frequencies. The results were applicable as these frequencies are mostly used in architectural acoustics and in most extant studies. It also made it easier to compare the results of this study with those of other common construction materials of the same density and, thus, to classify the results in line with the relevant rules.

Particleboards with medium and coarse particle sizes, P2D1 and P3D1, demonstrated superior noise absorption at high frequencies compared to commercial wood and plywood. However, noise absorbance at low frequencies was poor in all the samples. Small, medium, and large particle sizes of particleboards absorb more sound than wood and plywood at all frequency ranges except 125–250 Hz. The noise absorbance of those with small particle sizes, P1D1, P1D2, P1D3, and P1D4, were similar to that of plywood.

According to regulations [38], materials with an α_n_ of 0.90–1.00, 0.30–0.55, and 0.15–0.25 may be classified as Class A, Class D, and Class E absorbents, while those with an α_n_ of less than 0.15 cannot be classified. Except for low to medium frequencies of 125–2000 Hz, the medium and coarse particle size samples, specifically P2D1 and P3D1, could be categorized as Class A sound absorption particleboards.

Other experiments using coir fibre [39] and date palm [40] have also showed that an increase in thickness leads to an increase in the coefficient of sound absorption; hence, increasing the thickness of the board would enhance its sound absorption qualities.

## 4. Conclusions

The results demonstrate that OPF fibre-reinforced composites have high sound absorption capabilities in general. The bulk density and particle size of OPF composites have a considerable impact on their sound absorption performance. For particles of same size, the αn decreased with increasing density. The experiment showed that medium (P2D1) and coarse (P3D1) particle sizes exhibited superior sound absorption performance with an α_n_ of more than 0.95. OPF fibre-reinforced composites are good sound insulators at a wide range of frequencies and effectively absorb medium to high-frequency sounds. The acoustic findings for that type are appropriate for sound-absorbing insulating materials. This board exceeds commercial wood planks, common planks, and plywood used in buildings as non-structural panels to absorb sound. Finally, this study demonstrates that discarded oil palm fronds that pollute the environment may be used to create economically feasible and satisfactory sound absorption materials to replace synthetic fibres and commercial wood used in acoustic panel materials.

## Figures and Tables

**Table 1:** A review of recent research on the acoustic absorption qualities of several natural fibre materials.

Year	Fibres	Key Findings	References
2022	Sugarcane	The flow resistance and acoustic absorption coefficient increase as the fibre size decreases.	Mehrzad [14]
2021	Date palm	The statistical analysis revealed that the sample with 55 mm in thickness and 175 kg/m^3^ density had the optimum acoustic performance.	Taban [15]
2021	Coffee	The fibre size had no significant influence on the absorption coefficient, while the fibre ratio CS-CSS of 70:30 showed only a minor improvement over 50:50.	Abdi [16]
2021	Kenaf	The average absorption coefficient for samples with a density of 200 kg/m^3^ and a thickness of 45 mm was 0.96, 0.69, and 0.93, respectively, at 1 kHs. At 2 kHz, values of 0.93 are attainable with the same thickness and density.	Taban [17]
2020	Bagasse	The thickness of fibrous materials has a considerable influence on acoustic performance. The flow resistivity and acoustic absorption coefficient rise increase with material thickness.	Malawade [18]
2019	Coir	The sound absorption of thicker samples with constant densities was greater, particularly at lower frequencies (<1000 Hz).	Taban [19]
2018	Kenaf	The absorption coefficient for samples with a bulk density of 140–150 kg/m^3^ and a thickness of 25–30 mm is greater than 0.5 beginning at 500 Hz and can reach 0.85 on average above 1.5 kHz.	Putra [20]
2018	Pineapple	The average acoustic absorption coefficient for PALF is 0.9 above 2 kHz for a thickness of 20 mm and 0.8 above 1 kHz for a thickness of 30 mm for constant fibre density.	Putra [21]
2017	Broom	Sound absorption at lower frequencies is enhanced with increasing sample thickness, as would be expected for this type of material.	Berardi [22]
2017	Oil palm	Sound absorption performance can be improved by increasing sample thickness and optimizing fibre density.	Khai Hee Or [23]
2016	Sugarcane	The flow resistance and acoustic absorption coefficient increase as the fibre size decreases.	Othmani [24]

**Table 2:** Type of board manufactured.

Type of Particle Size	Sample Code	Target Density (g/cm^3^)	Particle Size (mm)	Pressing Pressure (Mpa)	Pressing Time (Min)	Pressing Temp (°C)	No. of Boards
Fine	P1D1	0.3	0.2 to 0.6	1.0	5	140	1
	P1D2	0.4					1
	P1D3	0.5					1
	P1D4	0.6					1
Medium	P2D1	0.3	1.0 to 2.0	1.0	5	140	1
	P2D2	0.4					1
	P2D3	0.5					1
	P2D4	0.6					1
Coarse	P3D1	0.3	2.38 to 4.76	1.0	5	140	1
	P3D2	0.4					1
	P3D3	0.5					1
	P3D4	0.6					1

**Table 3:** Coefficients of sound absorption (α) according to frequency.

Material	Sample Code	Thickness (mm)	Frequency					
			125	250	500	1000	2000	4000
OPF	P1D1	10	−0.02	−0.02	0.25	0.36	0.30	0.48
	P2D1	10	−0.03	−0.02	0.16	0.21	0.33	0.97
	P3D1	10	−0.04	−0.03	0.14	0.21	0.38	0.86
	P1D2	10	−0.03	−0.01	0.19	0.23	0.17	0.28
	P2D2	10	−0.02	−0.03	0.21	0.45	0.47	0.44
	P3D2	10	−0.03	−0.03	0.15	0.25	0.56	0.67
	P1D3	10	−0.02	−0.02	0.15	0.20	0.15	0.32
	P2D3	10	−0.04	−0.02	0.24	0.39	0.26	0.30
	P3D3	10	−0.03	−0.03	0.23	0.45	0.27	0.28
	P1D4	10	−0.05	−0.04	0.11	0.12	0.06	0.11
	P2D4	10	−0.03	−0.02	0.19	0.26	0.22	0.35
	P3D4	10	−0.02	−0.02	0.19	0.21	0.12	0.22
Wood [37]		25	0.19	0.14	0.09	0.06	0.06	0.05
Plywood [37]		9	0.28	0.22	0.17	0.09	0.1	0.11
OPF perforated [25]		12	NA	0.12	0.20	0.40	0.70	NA
Insulation classes [38]			-	-	E	D	D	A

## Data Availability

Not applicable.
